# Case Report: Reconstruction of the chest wall with titanium alloy plates after resection of a rare malignant spindle cell tumor of the sternum complicated by ankylosing spondylitis

**DOI:** 10.3389/fsurg.2026.1748981

**Published:** 2026-03-05

**Authors:** Xuhong Wang, Pengfei Zhu, Yunjie Zhang

**Affiliations:** 1 The First Clinical Medical College, Shandong University of Traditional Chinese Medicine, Shandong, China; 2 Department of Thoracic Surgery, Affiliated Hospital of Shandong University of Traditional Chinese Medicine, Shandong, China; 3 Department of Gastrointestinal and Hernia Surgery, Affiliated Hospital of Shandong University of Traditional Chinese Medicine, Shandong, China

**Keywords:** ankylosing spondylitis, case report, chest wall reconstruction, chest wall resection, malignant spindle cell tumor of the sternum, titanium alloy plates

## Abstract

**Background:**

Primary malignant sternal spindle cell tumors are clinically rare, and the aggressive nature leads to a large chest wall defect because of extended resection. To date, no cases of sternal malignant spindle cell tumor complicated by ankylosing spondylitis have been documented in the literature.

**Case presentation:**

We present a case of primary sternal malignant spindle cell tumor occurring in the setting of ankylosing spondylitis. Given the high risks associated with ankylosing spondylitis and the existing pathological fracture, preoperative biopsy was withheld after MDT evaluation. Intraoperative frozen section pathology confirmed malignant spindle cell tumor, and extended resection and chest wall reconstruction with a multi-point fixation strategy were subsequently completed. Postoperative staging was Enneking IIB (G2T2M0) with wide margins. The patient declined postoperative chemotherapy and underwent regular follow-up examinations. The patient recovered uneventfully without implant-related complications.

**Conclusion:**

This case report provides preliminary evidence that the combination of precise extended resection and chest wall reconstruction may achieve oncological radical cure in this specific patient. The procedure restored chest wall structure and function. These findings suggest the potential for favorable prognosis with this approach in similar complex cases of AS. This patient achieved 3-year disease-free survival. A complete dataset was provided including pulmonary function indices, exercise tolerance, and quality-of-life scores. These findings offer an important reference for clinical decision-making in similar complex cases.

## 1 Introduction

Primary sternal malignant spindle cell tumors are exceedingly rare. According to the multi-society joint guideline, the estimated incidence is no more than 0.018 per 100,000 people per year (1). Complete radical resection combined with chemotherapy constitutes the standard therapeutic protocol for this disease. This approach frequently leads to huge chest wall defects (2). Titanium alloy plates have gradually been used in complex chest wall reconstruction in recent years. However, their definitive application value is yet to be established in the highly complex and exceptionally rare scenario of sternal tumor complicated by ankylosing spondylitis(AS).

This case report presents three distinctive features: (1) This represents the first reported case of primary malignant spindle cell tumor of the sternum occurring concomitantly with AS. (2) We employed a novel multi-point fixation strategy, which balanced reconstructive rigidity with biocompatibility. (3) This case report provides systematic longitudinal data including pulmonary function, exercise tolerance, and quality-of-life metrics throughout 3 years post-operatively.

Notably, preoperative biopsy was withheld in this case, which stemmed from the specific pathophysiological constraints of AS. Additionally, the patient declined chemotherapy. These circumstances represented deviations from standard oncological protocols. Their clinical rationale and limitations will be critically discussed in the Discussion section.

## 2 Case presentation

**Figure 1:**
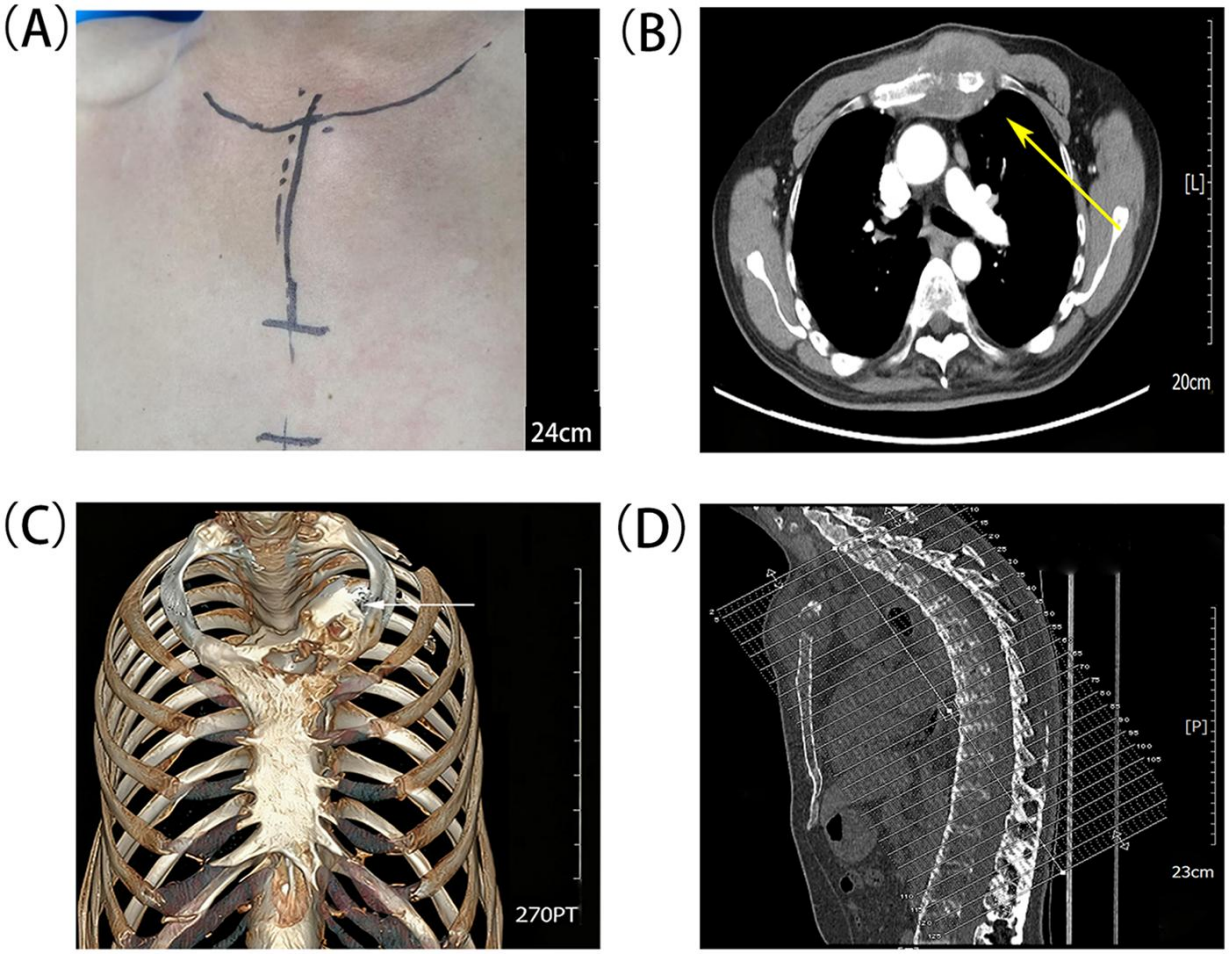
Preoperative computed tomography (CT) images of the sternal malignant tumor. **(A)** Clinical photograph of the anterior chest wall showing the surface appearance of the tumor lesion prior to surgery, with patient identifying features appropriately protected. **(B)** Axial contrast-enhanced CT image at the level of maximum tumor thickness showing an expansile destructive lesion in the manubrium sterni with a large soft tissue mass and pathological fracture(yellow arrow). **(C)** 3D CT reconstruction image demonstrating the extent of tumor invasion, which extended beyond the left sternocostal joint, invading the medial clavicular head and the first and second costal cartilages(white arrow). **(D)** Sagittal CT image revealing AS, providing an important reference for preoperative surgical risk assessment and surgical planning.

The patient, a 52-year-old male, was admitted to our hospital with a chest mass and pain for more than 7 years. A clinical photograph of the anterior chest wall showed the surface appearance of the tumor prior to surgery (Figure 1A). The contrast-enhanced CT and 3D reconstruction revealed bony destruction of the sternal manubrium with a pathological fracture and an adjacent soft tissue mass (Figures 1B,C). Diffuse bony fusion involving the spine and thoracic cage with observed fusion of thoracic facet joints and ossification of surrounding ligaments was consistent with AS (Figure 1D). After discussion by the MDT composed of thoracic surgery, medical oncology, radiology, pathology, and anesthesiology departments, it was determined that complete tumor resection was feasible based on imaging features such as no mediastinal invasion or distant metastasis. Preoperative biopsy was initially considered. However, given the aforementioned sternal pathology, the MDT opted to proceed directly with surgery. Intraoperative frozen-section pathology was employed to ascertain histological diagnosis. A multi-point fixation strategy was adopted with customized titanium alloy plates to match the thoracic cage morphology.

Intraoperatively, exploration revealed the tumor was situated in the sternal manubrium, extending superiorly to the sternal notch, inferiorly to the sternal angle, anteriorly infiltrating the pectoralis muscles, and bilaterally reaching the sternocostal joints. It further extended beyond the left sternocostal joint to involve the medial end of the clavicle as well as the first and second costal cartilages. The tumor, bilateral sternocostal joints and the inferior edges of bilateral first and second costal cartilages were resected. The resection on the left side included the medial end of the clavicle, and the lower boundary involved transverse transection of the upper segment of the sternal body. During the operation, the involved area of the left pectoralis major muscle demonstrated adhesive infiltration. However, intraoperative palpation confirmed a separable plane between the lesion and the main muscle fibers as well as the thoracoacromial vascular pedicle. Therefore, we performed an extended resection of the superficially involved portion of the left pectoralis major muscle, preserving the thoracoacromial neurovascular pedicle. We designed partial resection of the left pectoralis major with preservation of the vascular pedicle. This procedure was utilized for subsequent soft tissue coverage. The right pectoralis major muscle was not involved by tumor. We performed partial release of its costal attachments. Subsequently, the tumor mass, the planned preservation zone of the left pectoralis major muscle, and the surgical margin were submitted for intraoperative frozen section pathology. Five consecutive samples were taken from the preservation zone along the longitudinal axis of muscle fibers at 1 cm intervals. These samples included the vascular pedicle root. The surgical margin specimens included anterior and posterior soft tissue margins. The anterior margins were obtained at the extended pectoralis major resection site. Intraoperative frozen pathology confirmed a malignant spindle-cell tumor, with no tumor cells identified in the anterior and posterior margins, as well as in the serial samples from the left pectoralis major base. Specific results were awaiting confirmation by routine pathology. Osseous components included superior and inferior margins and bilateral margins. These structures were resected with a ≥4 cm macroscopic safety margin. Superior and inferior margins were located at the sternal notch level and the transverse section of the upper sternal body. Bilateral margins included bilateral first and second sternocostal joints, costal cartilage regions, and the residual medial end of the left clavicle. These specimens were sent for routine pathology. The resection scope was approximately 11.5 cm × 9.5 cm × 3.5 cm. No tumor infiltration was identified in the anterior mediastinum, lungs, or lymph nodes. The continuity of the thoracic cage was completely disrupted at the level of the thoracic inlet, necessitating rigid chest wall reconstruction.

**Figure 2:**
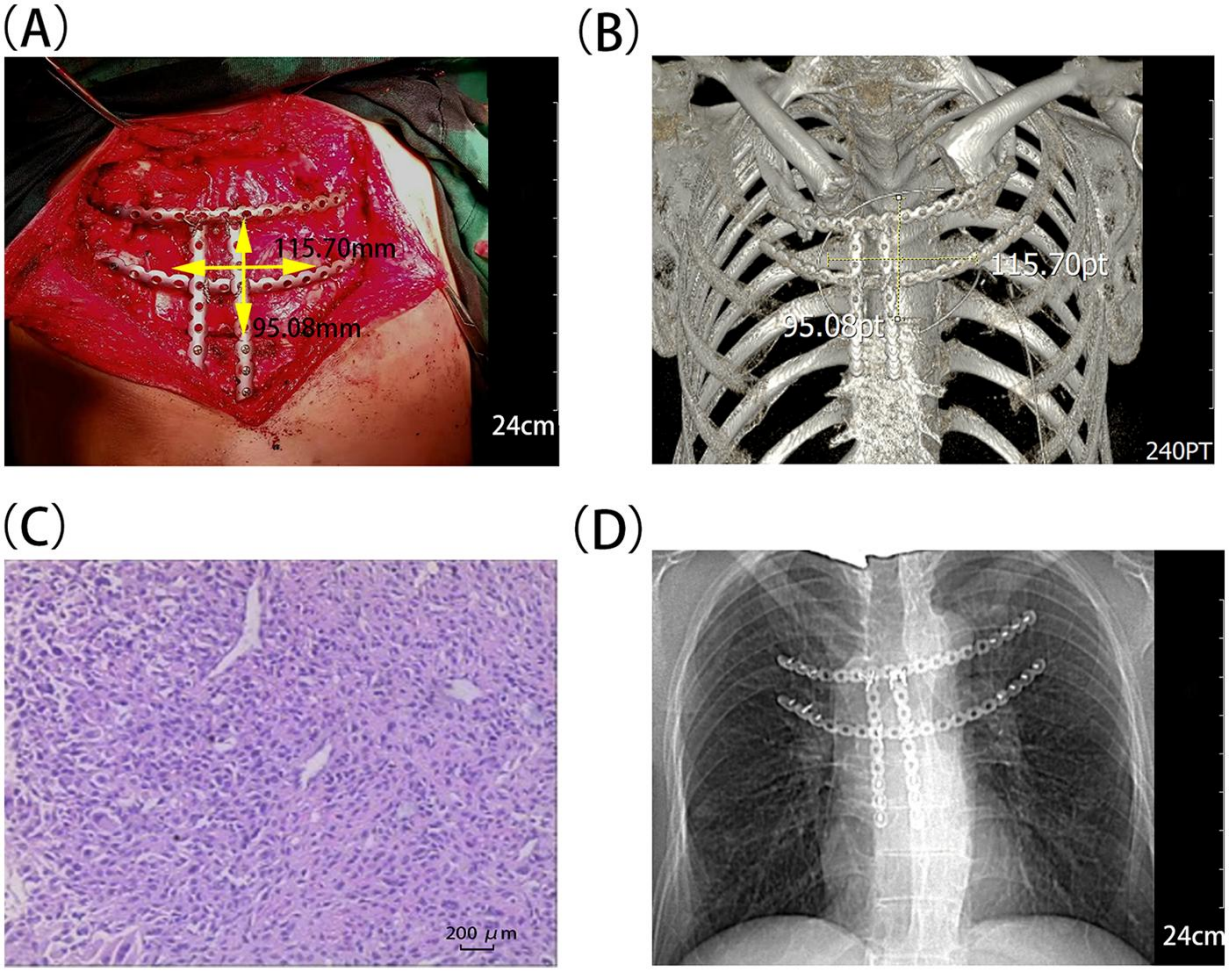
Intraoperative photograph and postoperative imaging after chest wall reconstruction. **(A)** Intraoperative photograph following *en bloc* tumor resection showing a chest wall defect measuring approximately 11.5 cm × 9.5 cm × 3.5 cm, with complete disruption of the thoracic cage continuity at the thoracic inlet. Chest wall reconstruction is being performed with titanium alloy plates and multi-point fixation. White dashed lines mark the extent of resection, including the sternal manubrium and body, bilateral first-second costal cartilages, and left medial clavicle. **(B)** Postoperative 3D CT reconstruction showing the precise reconstruction scope, including the medial side of the left clavicle, bilateral 1st to 2nd costal cartilages, and upper segment of the sternal body. The reconstructed osseous scaffold restored the continuity of the thoracic cage. **(C)** Postoperative pathological examination image showing (sternal) spindle cell malignant tumor. **(D)** Postoperative chest x-ray confirming titanium alloy plates and screws in good position, with no signs of internal fixation displacement.

The malleable titanium alloy plates were manually contoured to match the anatomical curvature of the patient's anterior chest wall based on real-time anatomical landmarks and imaging features. The titanium alloy plates were then attached to the bone surface with minor adjustments, ensuring they closely fit the surfaces of the costal cartilage and sternal body. Given the aberrant bone metabolism and thoracic rigidity in AS, we intraoperatively introduced three targeted modifications to the fixation protocol: (1) Screws were inserted at a 30° angle relative to the long axis of the costal cartilage; (2) Bicortical fixation was performed at the transverse end of the sternal body; (3) Additional steel-wire cerclage was applied at the junctions of the titanium plates (Figure 2A).

Under direct vision, no chest wall collapse or paradoxical respiration in the defect area was observed when the anesthesiologist increased the airway pressure. Absorbable polyglycolic acid patches were applied to cover all titanium plates. Given confirmation of negative margins, we performed bilateral pectoralis major muscle advancement. On the right side, the muscle was released from its costal origin and advanced toward the midline. On the left side, partial resection was performed with preservation of the thoracoacromial vascular pedicle and the residual stump was advanced toward the midline. Bilateral interrupted suture fixation was performed to complete soft tissue layer reconstruction.

The postoperative respiratory physiotherapy was instituted immediately. Postoperative 3D CT reconstruction demonstrated well-fixed titanium alloy plates. These plates were located on the residual bilateral first and second costal cartilages. Four screws were implanted in each left-side plate. Three screws were implanted in each right-side plate. The sternal body plates were placed parallel to the longitudinal axis. Three screws were placed on each side (Figure 2B). The postoperative pathological report disclosed a spindle-cell malignant tumor of the sternum that had invaded the adjacent striated muscle and bone tissue, with tumor thrombi found in blood vessels (Figure 2C). According to the Enneking staging system, malignant spindle cell tumors with extracompartmental extension were classified as Stage IIB (G2T2M0). Wide surgical margins (≥4 cm) were achieved according to the requirements of this staging system. Immunohistochemistry showed: CK5/6 (positive, focal), CD34 (positive, vascular), S-100 (negative), Desmin (negative), SATB2 (negative), SMA (negative), Ki67 (positive, 20%), Bcl-2 (negative), STAT6 (negative), Beta-catenin (positive, cytoplasmic). No tumor cells were detected in any surgical margins or in serial sections from the base of the left pectoralis major muscle. The patient declined postoperative chemotherapy.

The three-month postoperative follow-up demonstrated clear visualization of the steel wire connection structures between titanium alloy plates, with no evidence of wire fracture or loosening. The titanium alloy plates were in close apposition to the bone surface with stable fixation (Figure 2D). Thoracic cage stability was good, with no paradoxical respiratory movements.

During the 3-year postoperative follow-up, no tumor recurrence or metastasis was observed, and the chest wall stability remained good. During this period, there was no chronic pain, and respiratory function remained normal. Additionally, daily activities were not limited, and the quality of life was excellent during the 3-year postoperative follow-up (Table 1).

**Table 1:** Preoperative and 3-year postoperative follow-up data.

Category	Specific Parameter	Preoperative	Postoperative Day 1	Postoperative Week 1	6 Months	1 Year	2 Years	3 Years
Symptom Assessment (Quantification)	Pain Score (NRS)	7	3	0	0	0	0	0
	Dyspnea Score (mMRC)	3	2	1	0	0	0	0
Pulmonary Function Indicators	Forced Vital Capacity (FVC): Measured Value/ Predicted Value (%)	95	-	85	90	95	95	95
	Forced Expiratory Volume in 1 Second (FEV1): Measured Value/ Predicted Value (%)	95	-	84	86	95	95	95
	Oxygen Saturation (SpO_2_)	93	91	96	99	99	99	99
Physical Examination Indicators	Respiratory Sounds (Auscultation)	Diminished, accompanied by moist rales	Diminished, accompanied by moist rales	Mildly diminished	Clear	Clear	Clear	Clear
Functional Recovery Indicators	Exercise Tolerance (6-Minute Walk Test)	300	-	280	400	430	450	450
	Weight-Bearing Exercise: Volume 0 kg (No Weight Bearing)—Daily Weight Bearing (5 kg Object Lifting)	1	0	0	3.5	5	5	5
Quality of Life	EORTC QLQ-C30 Physical Function: Standardized via Formula, 0–100 Points	47	33	33	67	73	78	80
	EORTC QLQ-C30 Global Health Status: Standardized via Formula, 0–100 Points	33	17	33	50	67	75	75
	Karnofsky Performance Status (KPS): 0–100 Points	65	45	50	70	75	78	82

## 3 Discussion

Primary malignant sternal spindle cell tumors are exceedingly rare. This rarity results in a lack of standardized diagnostic and therapeutic algorithms (1) In the present case, the final diagnosis was an Enneking Stage IIB (G2T2M0) tumor. This stage mandated extensive resection to achieve adequate oncological margins. However, AS was not merely a comorbidity. Its specific pathophysiology influenced both the diagnostic approach and the reconstruction strategy.

Preoperative biopsy is the standard protocol for suspected primary bone sarcoma. However, it was withheld following MDT consensus. This decision was based on the pathophysiological environment of AS. The patient presented with a pathological fracture of the sternal manubrium and diffuse bony fusion of the thoracic cage. Meta-analyses and nationwide registry-based cohort studies have indicated that patients with AS exhibit abnormal bone metabolism and increased bone fragility (3), with a 20% elevated risk of non-vertebral fractures compared with the general population (IRR=1.2, 95%CI 1.1–1.3) (4). Preoperative biopsy posed risks of fracture displacement and vascular injury and compromised tolerance to potential pneumothorax or hemothorax. Intraoperative frozen-section pathology was employed to ascertain malignant characteristics. This approach guided extended resection. Final bony margin status was determined by postoperative routine pathology. To achieve the wide surgical margins required for this Enneking Stage IIB tumor, we performed an extensive resection that resulted in a complex defect measuring 11.5 cm in maximal dimension and spanning multiple anatomical units, thereby completely disrupting thoracic cage continuity at the thoracic inlet (Figure 2A) and mandating rigid reconstruction per international consensus (5). This case was distinct from simple sternectomy performed for post-sternotomy osteomyelitis. The latter preserves bilateral costal-cartilage attachments, thereby maintaining the integrity of the thoracic ring. Formal rigid reconstruction is therefore rarely required. Based on comprehensive MDT assessment of operative duration and patient tolerance, the team decided to proceed with immediate reconstruction.

However, in the setting of AS, reconstruction also faces specific pathophysiological constraints. First, as previously described, poor bone quality poses a high risk of screw loosening or costal cartilage fragmentation for conventional fixation techniques. Second, the thoracic rigidity induced by ankylosing spondylitis reduces chest wall compliance. This creates a restrictive ventilatory physiology and increases perioperative risks. According to the ERS/ATS technical standard on interpretive strategies for routine lung function tests (6), the reporting format of “measured/predicted (%)” eliminates individual variability, conferring significant value for follow-up and multi-center efficacy comparisons. In this patient, the preoperative FVC appeared to be within the normal reference range. However, as the global lung function initiative average equation (7) are constructed from healthy population databases that do not incorporate the restrictive ventilatory pathophysiology characteristic of AS. Consequently, in AS patients, even with seemingly normal pulmonary function data, actual respiratory reserve and exercise tolerance may be overestimated. The patients with pre-existing restrictive physiological conditions have a greater dependence on chest wall stability (5). These specific constraints associated with AS necessitated targeted modifications to reconstruction materials and fixation strategies.

Contemporary complex chest wall reconstruction advocates a composite strategy integrating rigid and nonrigid elements rather than selecting a single material (5). The MDT defined three criteria including adequate mechanical strength, excellent biocompatibility and minor interference with postoperative imaging. For the rigid scaffold component, biodegradable materials such as BioBridge Rib Prosthesis (8) and acellular dermal matrix (9) possess strong anti-infection ability and serve as scaffolds to promote tissue ingrowth, they have insufficient mechanical strength and carry risks of thoracic deformity (10–12). Among the remaining alternatives, three-dimensional-printed customized prostheses enable personalized design but lack intraoperative adjustability. Nevertheless, this technology is still in the developmental stage, and its production cycle affects surgical scheduling. In comparison, we ultimately selected titanium alloy plates as the rigid scaffold component. They show excellent anatomical adaptability for large-scale complex defects with high hardness and ease of shaping (13). Titanium alloy has good biocompatibility (8). Its use can reduce the incidence of rejection reactions, allergic reactions, and postoperative infection (9). They exhibit minimal imaging interference facilitating long term surveillance of AS and tumor recurrence.

Given bone fragility and thoracic rigidity, we adopted a multi-point fixation strategy. We implemented three modifications to conventional techniques. (1) Screws were placed along the costal cartilage at a 30° angle to its long axis. This approach created elastic clamping rather than rigid locking to prevent fragmentation. (2) During fixation of the transverse end of the sternal body, screws were inserted through both cortices of the sternum to achieve bicortical fixation and maintain force transmission. (3) The titanium alloy plates were fixed with steel wires at the connection sites. After passing the steel wires through the holes of the titanium alloy plates, the surgeon tightened and secured the steel wires to allow the titanium alloy plates to disperse the stress. All steel wire knots were buried in the edge grooves of the titanium alloy plates or folded toward the bone surface, with appropriate tension maintained to avoid irritating muscles and soft tissues (Figure 2A). The composite reconstructive strategy included intraoperative coverage of titanium plates with absorbable polyglycolic acid patches and bilateral pectoralis major muscle advancement with preservation of the thoracoacromial vascular pedicle.

This case report provided long-term diagnosis and treatment data for this extremely rare pathological tumor type. (1) Parallel recovery of FVC and FEV1 was higher than that reported in the literature (14). This was likely attributed to improved efficiency of diaphragmatic compensation following restoration of chest wall stability with the multi-point fixation strategy in this patient with AS. (2) Quality of life and activity-related indices recovered to normal levels for his age. (3) No tumor recurrence or implant-related complications were observed during long-term follow up (15).

Despite favorable clinical outcomes, we acknowledge important methodological limitations that warrant transparent discussion. First, preoperative biopsy was withheld, representing a deviation from standard protocols. However, this decision was based on MDT consensus given the high risks associated with pathological fracture in AS and should not be generalized. Second, the patient declined adjuvant chemotherapy. The current disease-free survival may be attributed to wide excision and regular follow-up examinations but this single modality treatment approach may not be generalizable to standard patient populations. Third, comprehensive preoperative respiratory baseline data were lacking due to preoperative chest wall pain and thoracic rigidity secondary to AS. Fourth, the long-term safety of titanium alloy implants in AS remains uncertain beyond the current follow-up period. AS may increase the risk of implant-related complications through the following mechanisms: (1) Stress shielding induced bone resorption and potential late screw loosening due to aberrant bone metabolism associated with AS (16). (2) Potential inhibition of bone integration by chronic inflammatory factors (17). (3) Macrophage inflammatory response and secondary osteolysis induced by release of titanium particles and ions (18).

## 4 Conclusions

This case represents the first report of primary malignant spindle cell tumor of the sternum occurring concomitantly with AS. AS as a significant comorbidity influenced the entire course of diagnosis and treatment: Pathological fracture and thoracic rigidity prompted the MDT to withhold preoperative biopsy. Bone fragility drove innovative modifications to the multi-point fixation strategy. Although the patient declined chemotherapy, wide excision and regular follow-up examinations achieved 3-year disease-free survival without implant-related complications. This case provides a preliminary reference for similar complex cases.

## 5 Patient perspective

Preoperatively, the patient suffered from chest wall pain and AS During the three-year postoperative period, he reported significant improvement in quality of life. The personalized reconstruction technique ensured both functional integrity of the chest wall and acceptable cosmetic outcome, which he considered a key factor in his psychological recovery. The patient continues to undergo regular oncological monitoring, with no current signs of cancer recurrence.

## Data Availability

The original contributions presented in the study are included in the article/Supplementary Material, further inquiries can be directed to the corresponding author.
